# FoxO and Stress Responses in the Cnidarian *Hydra vulgaris*

**DOI:** 10.1371/journal.pone.0011686

**Published:** 2010-07-21

**Authors:** Diane Bridge, Alexander G. Theofiles, Rebecca L. Holler, Emily Marcinkevicius, Robert E. Steele, Daniel E. Martínez

**Affiliations:** 1 Department of Biology, Elizabethtown College, Elizabethtown, Pennsylvania, United States of America; 2 Department of Biology, Pomona College, Claremont, California, United States of America; 3 Department of Biological Chemistry and the Developmental Biology Center, University of California Irvine, Irvine, California, United States of America; Michigan State University, United States of America

## Abstract

**Background:**

In the face of changing environmental conditions, the mechanisms underlying stress responses in diverse organisms are of increasing interest. In vertebrates, *Drosophila*, and *Caenorhabditis elegans*, FoxO transcription factors mediate cellular responses to stress, including oxidative stress and dietary restriction. Although FoxO genes have been identified in early-arising animal lineages including sponges and cnidarians, little is known about their roles in these organisms.

**Methods/Principal Findings:**

We have examined the regulation of FoxO activity in members of the well-studied cnidarian genus *Hydra*. We find that *Hydra FoxO* is expressed at high levels in cells of the interstitial lineage, a cell lineage that includes multipotent stem cells that give rise to neurons, stinging cells, secretory cells and gametes. Using transgenic *Hydra* that express a FoxO-GFP fusion protein in cells of the interstitial lineage, we have determined that heat shock causes localization of the fusion protein to the nucleus. Our results also provide evidence that, as in bilaterian animals, *Hydra* FoxO activity is regulated by both Akt and JNK kinases.

**Conclusions:**

These findings imply that basic mechanisms of FoxO regulation arose before the evolution of bilaterians and raise the possibility that FoxO is involved in stress responses of other cnidarian species, including corals.

## Introduction

In bilaterian animals, members of the FoxO family of transcription factors are well-known for their roles in cellular responses to environmental and physiological stress. A single FoxO gene is present in *Drosophila* (*dFOXO*) and *C. elegans* (*daf-16*), and four are present in mice and humans (*FoxO1*, *FoxO3*, *FoxO4*, and *FoxO6*). In *Drosophila*, *C. elegans*, and mammalian cells, FoxO proteins increase resistance to oxidative stress [1]–[4]. Transcription of FoxO target genes increases under low nutrient conditions in *Drosophila*, *C. elegans*, and mammals [4]–[6] and during heat shock in *C. elegans* [7]. FoxO proteins mediate diverse cellular responses to stress. In *Drosophila*, *C. elegans* and mammals, they increase levels of enzymes that detoxify reactive oxygen species (ROS) [1]–[3], [8]. During starvation, FoxO proteins induce autophagy in mouse skeletal muscle and cardiomyocytes, permitting recycling of cellular components [9]–[11]. dFOXO similarly induces autophagy in the fat body of *Drosophila* undergoing dietary restriction [12]. In *C. elegans* and mammals, FoxO proteins increase resistance to DNA damage [13]–[15]. In *C. elegans*, DAF-16 regulates transcription of genes involved in pathogen resistance and acts together with the transcription factor heat shock factor 1 to upregulate transcription of small heat shock protein genes [7], [16].

Activity of FoxO proteins is regulated by post-transcriptional modification, including phosphorylation. In response to insulin/IGF-1 and other growth factors, Akt and the related serum- and glucocorticoid-inducible kinase (SGK) phosphorylate FoxO proteins (with the exception of FoxO6) at three conserved sites. These phosphorylations promote FoxO binding to 14-3-3 proteins and localization to the cytoplasm [17]–[20], where FoxO proteins cannot regulate transcription. In contrast, in response to stress the c-Jun N-terminal kinase (JNK) pathway causes FoxO nuclear localization and increased transcriptional activity [21]–[23]. JNK has been shown to phosphorylate DAF-16 and FoxO4 directly [21], [22]. JNK-dependent nuclear localization of mammalian FoxO proteins may also involve phosphorylation of 14-3-3 proteins followed by their disassociation from FoxO proteins [24].

A substantial amount is known about the role of FoxO proteins in the stress responses of bilaterian animals. However FoxO genes have also been identified in members of earlier-evolving metazoan lineages, such as sponges [25] and cnidarians [26], [27], and the functions of FoxO proteins in these groups remain unknown. The physiology of stress responses in cnidarian species is of increasing interest, given the effects of climate change and pollution on aquatic habitats, e.g. [28], [29]. To address the question of whether FoxO proteins are involved in stress responses in cnidarians, we focused on the cnidarian genus *Hydra*. The *Hydra magnipapillata* genome has been sequenced [30], and transgenic *Hydra* can be produced [31]. In addition, the physiological response to heat shock in *Hydra* has been examined in some detail [32]–[34].

Cell and tissue dynamics in *Hydra* species are well characterized [35]. The cylindrical body of the adult *Hydra* polyp consists of two tissue layers, an ectoderm and an endoderm. At one end of the body is a mouth surrounded by a ring of tentacles. At the other end is a basal disk by which the animal adheres to the substrate. In the adult, cells divide continuously in the body column. Cell division in the body column causes displacement of epithelial cells into the tentacles and basal disk [36], [37], where cell cycle arrest and terminal differentiation occur [38]. Adult *Hydra* routinely reproduce asexually, with cells from the body column displaced into buds. The continuous growth and production of buds by adult *Hydra* can be explained in part by the presence of three types of multipotent stem cells. These are ectodermal stem cells, endodermal stem cells, and interstitial stem cells. Interstitial stem cells are located between epithelial cells in the ectodermal layer and give rise to neurons, secretory cells, gametes, and nematocytes, the stinging cells unique to cnidarians [39]. We have characterized the expression of *FoxO* in *H. vulgaris* and the closely related *H. magnipapillata* and have examined cellular localization of a FoxO-GFP fusion protein in transgenic animals. We find evidence for significant parallels in regulation of FoxO between *Hydra* and bilaterian animals.

## Results

### A single FoxO gene is present in *H. magnipapillata*

**Figure 1:**
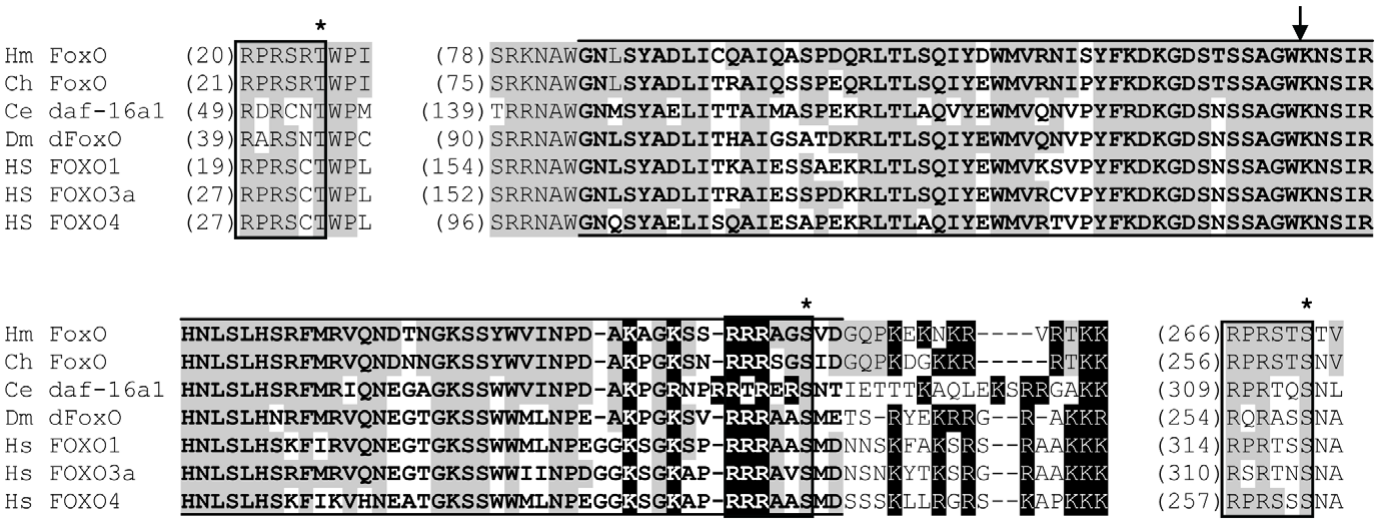
Conserved portions of the predicted *H. magnipapillata* FoxO protein aligned with other FoxO protein sequences. Akt/SGK phosphorylation motifs are enclosed in boxes, with asterisks above phosphorylated residues. The arrow indicates the location of the intron present in *H. magnipapillata* FoxO and the other sequences shown. Lines above and below the sequence indicate the forkhead domain. Basic amino acids characteristic of the nuclear localization domain are highlighted in black. Amino acids identical in *H. magnipapillata* FoxO and another sequence are shaded.

Searches of the *H. magnipapillata* genome identified a single FoxO gene. The predicted *H. magnipapillata* FoxO protein (Figure 1) includes the forkhead winged helix domain characteristic of Fox proteins. A single intron is located within the region encoding the forkhead domain. An intron is present at the same location in FoxO genes from multiple other species, as well as in members of some other Fox gene families [25]. As in known FoxO proteins (with the exception of FoxO6), three consensus Akt/SGK phosphorylation sites are present [4], [40]–[44]. Stretches of basic amino acids overlapping the end of the forkhead domain match the consensus sequence for a bipartite nuclear localization signal [45].

**Figure 2:**
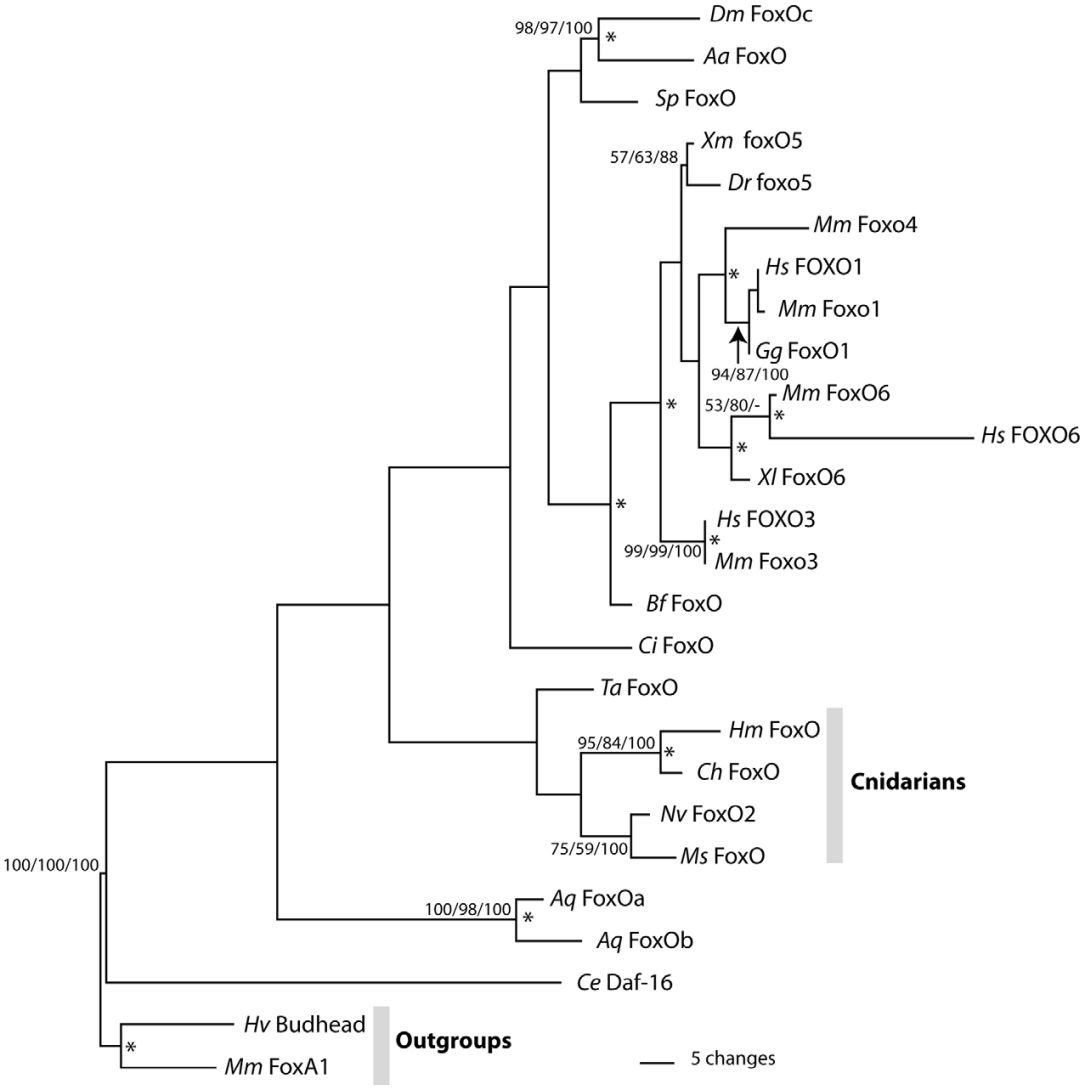
Results of phylogenetic analyses. Maximum parsimony phylogram of selected FoxO proteins rooted using *Mus musculus* FoxA1. Numbers at nodes are bootstrap support values calculated by 1000 replicates of Maximum Parsimony/Maximum Likelihood/Neighbor Joining. Bootstrap values under 50 are not shown. Asterisks at nodes indicate Bayesian PP greater than 95%. Species name abbreviations: *Aa: Aedes aegypti*; *Aq: Amphimedon queenslandica*; *Bf: Branchiostoma floridae*; *Ce: Caenorhabditis elegans*; *Ch: Clytia hemisphaerica*; *Ci: Ciona intestinalis*; *Dm: Drosophila melanogaster*; *Dr: Danio rerio*; *Gg: Gallus gallus*; *Hm: Hydra magnipapillata*; *Hs: Homo sapiens*; *Hv: Hydra vulgaris*; *Mm: Mus musculus*; *Ms: Metridium senile*; *Nv: Nematostella vectensis*; *Sp: Strongylocentrotus purpuratus*; *Ta: Trichoplax adhaerens*; *Xl: Xenopus laevis*; and *Xm: Xiphophorus maculatus*.

Phylogenetic analyses based on the predicted amino acid sequence of the forkhead domain were performed to confirm that the *Hydra* gene is a member of the FoxO family. An initial phylogenetic analysis included representatives from mouse of all known Fox gene families. This analysis also included Fox genes from *Clytia hemisphaerica* [27], a species that, like *Hydra*, belongs to the cnidarian class Hydrozoa. We found that the *H. magnipapillata* gene groups with FoxO genes as expected (data not shown). Phylogenetic analyses including sequences of FoxO forkhead domains from diverse animals were also performed (Figure 2). BLAST searches of the genome of *Trichoplax adherens*, a member of the early-evolving animal phylum Placozoa, identified a FoxO gene which was included in analyses. Results of phylogenetic analyses place *H. magnipapillata FoxO* together with FoxO genes from other cnidarian species. The results imply that *H. magnipapillata FoxO* is the ortholog of *daf-16* and *Drosophila dFOXO*. Vertebrate FoxO sequences form a group within the FoxO family, confirming that duplication of FoxO genes has occurred within the vertebrate lineage.

### FoxO is expressed in interstitial cells

*Hydra* interstitial cells are located between epithelial cells, mainly within the ectoderm. They are present in the body column, but not in the tentacles or basal disk [39]. Interstitial cells include multipotent interstitial stem cells, committed differentiation intermediates derived from them, and unipotent stem cells, also derived from the multipotent interstitial stem cells, which produce eggs or sperm [39]. Interstitial cells give rise to neurons, secretory cells, nematocytes, and gametes [46], [47]. During the process of nematocyte formation, cells divide to form nests of nematoblasts connected by cytoplasmic bridges [48]. Cells within the nests then differentiate, separate, and migrate to their final locations. Like interstitial cells, nematoblasts are found in the ectoderm of the body column. Neurons also differentiate within the body column but are present in both tissue layers. Secretory cells are found only in the endoderm. Cells which will give rise to sperm migrate within the body column and accumulate under the ectoderm in rounded structures known as testes [49]. Cells with the potential to form eggs proliferate and form a mass under the ectoderm [50]. One oocyte develops within the mass, while the remaining cells transfer cytoplasm to the developing oocyte, initiate apoptosis, and are phagocytosed by the oocyte [50]–[52].

**Figure 3:**
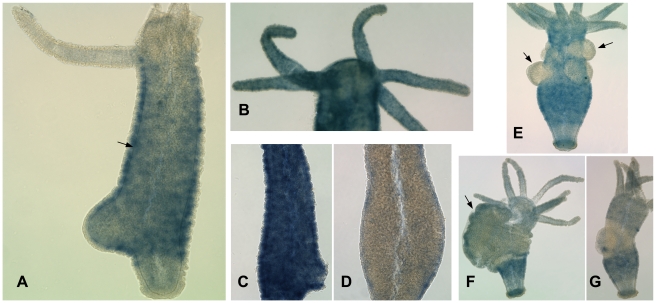
Results of whole mount *in situ* hybridization. A) Expression of *FoxO* mRNA in adult *H. magnipapillata*. The arrow indicates the border between ectoderm and endoderm. B) Adult *H. magnipapillata* with longer staining reaction, showing punctuate staining in the tentacles. C) Body column of control *H. magnipapillata*. D) Body column of HU-treated *H. magnipapillata*. E) *H. vulgaris* with testes, indicated by arrows. F) *H. vulgaris* with developing egg, indicated by an arrow. G) *H. vulgaris* following egg extrusion.

Whole mount in situ hybridization detected *FoxO* mRNA in cells of the interstitial cell lineage, with expression stronger in the body column than in the tentacles, head, or basal disk (Figure 3A). Cells expressing *FoxO* are present in the ectoderm (Figure 3A), indicating that *FoxO* is expressed in interstitial cells, nematoblasts, and/or differentiating neurons. Fainter, punctuate expression in tentacle ectoderm (Figure 3B) suggests that it is also expressed at lower levels in nematocytes and/or neurons.

Because interstitial cells divide more rapidly than epithelial cells, their numbers can be differentially reduced by treatment of *Hydra* polyps with hydroxyurea (HU) [53]. To confirm that *FoxO* is expressed in interstitial cells, we treated *H. magnipapillata* with HU. Counting cells from macerated control and treated animals showed that treatment reduced the ratio of interstitial cells to total cells present by 85 to 90%, depending on the experiment, but did not reduce the ratio of nematoblasts, nematocytes, or neurons to total cells. (In control animals, we found that interstitial cells constituted 22 to 29% of total cells, depending on the experiment. These percentages are consistent with previous results [53], [54].) In situ hybridization showed substantially decreased *FoxO* expression in HU-treated compared to control animals (Figure 3C, D). Our results indicate that *FoxO* is expressed in interstitial cells, although they do not preclude the possibility that it is also expressed at lower levels in epithelial cells.

We also examined *FoxO* expression during spermatogenesis and oogenesis using the *H. vulgaris* AEP strain, which readily reproduces sexually in the laboratory. No expression was detected in testes, where proliferation of spermatogonia and their differentiation into sperm take place [55] (Figure 3E). A low level of *FoxO* expression was found in developing oocytes (Figure 3F). Following oogenesis, the former egg field is depleted of *FoxO*-expressing cells (Figure 3G).

### FoxO-GFP localization is affected by PI3K-mediated signaling, heat shock, and JNK

**Figure 4:**
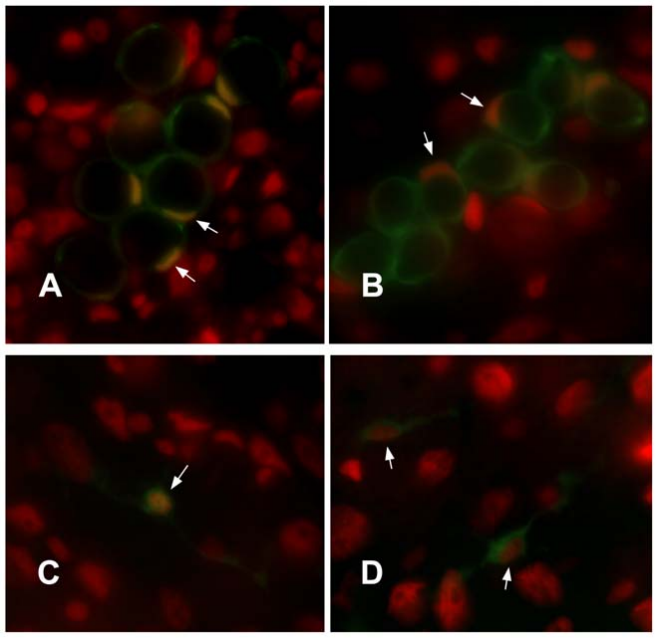
*Hydra* FoxO-GFP localization in cells of the interstitial lineage. (A, C) Nuclear localization. (B, D) Cytoplasmic localization. DAPI-stained nuclei are false-colored red, FoxO-GFP green, areas of overlap yellow. A, B) Late stage nematoblasts. The nucleus is crescent-shaped as a result of being pushed to the side of the cell by the developing nematocyst capsule. C, D) Neurons. Arrows indicate nuclei.

To examine the regulation of FoxO cellular localization in *Hydra*, we produced lines of transgenic *H. vulgaris* which express a FoxO-GFP fusion protein under the control of a *Hydra* β-actin promoter. *Hydra* ectodermal, endodermal, and interstitial lineage cells represent separate cellular compartments, replenished by separate stem cells. Embryo microinjection can therefore produce animals with stably transgenic cells of one or more of the three cell lineages [31]. Because in situ hybridization indicated that *FoxO* is expressed at high levels in cells of the interstitial cell lineage, we examined protein localization primarily in transgenic animals that expressed the FoxO-GFP protein in cells of the interstitial lineage (Figure 4). In interstitial lineage cells, the *Hydra* β-actin promoter used drives expression in stenotele nematocytes, precursors to stenoteles, and ganglionic neurons (R.E. Steele et al., unpublished information). We characterized FoxO-GFP localization in these cell types.

**Figure 5:**
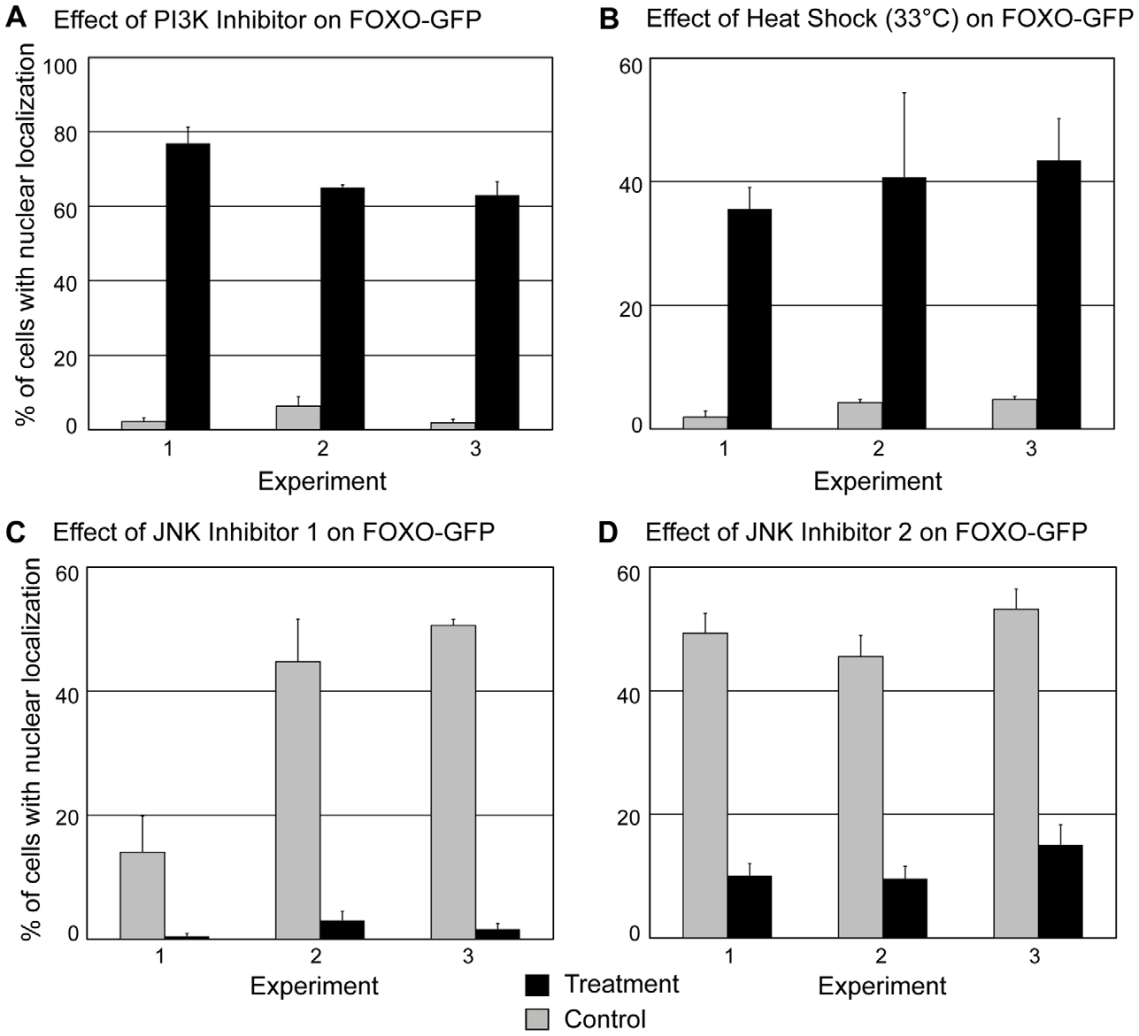
Effects of experimental treatments on FoxO-GFP localization. Localization was examined in stenotele nematocytes, nematoblasts which are precursors to stenoteles, and ganglionic neurons. A) Effects of PI3K inhibitor treatment—1 hour incubation in 40 µM LY294002. N≥69 cells examined per animal. B) Effects of heat shock—90 minutes at 33°C. N≥73 cells examined per animal. C) Effects of heat shock on animals treated with JNK inhibitor. Control and treatment animals were subject to heat shock. Treatment involved 24 hour incubation in 2.5 µM SP60025 prior to heat shock in 2.5 µM SP60025. N≥55 cells examined per animal. D) Effects of heat shock on animals treated with JNK inhibitor. Control and treated animals were subjected to heat shock. Treatment involved 24 hour incubation in 2 µM AS601245 prior to heat shock in 2 µM AS601245. N≥56 cells examined per animal.

In other organisms studied, insulin/IGF-1 signaling acts through phosphoinositide 3-kinases (PI3K), Akt, and SGK. Phosphorylation of FoxO by Akt and SGK promotes its cytoplasmic localization, reducing FoxO transcriptional activity [56]. The conserved consensus Akt/SGK phosphorylation sites present in the predicted *H. magnipapillata* FoxO protein suggest that Akt and/or SGK may be involved in regulating *Hydra* FoxO activity. Because PI3K activity leads to activation of Akt and SGK, we treated transgenic animals with an inhibitor of PI3K, LY294002 [57] to address the effect of decreased Akt and SGK activity on *Hydra* FoxO localization. We found that inhibitor treatment significantly increased the percentage of interstitial lineage cells showing nuclear localization of FoxO-GFP (Figure 5A), suggesting that Akt and/or SGK activity promote the cytoplasmic localization of *Hydra* FoxO.

To determine whether dietary restriction leads to FoxO nuclear localization in *Hydra*, we examined FoxO-GFP localization in transgenic animals that were fed daily for two weeks and then starved for two, three, four, or ten days. We found no significant difference in the percentage of interstitial lineage cells showing fusion protein nuclear localization between control animals and starved animals (N≥38 cells examined per animal, *P*≥0.1 in all cases).

To address the effect of heat shock on *Hydra* FoxO localization, transgenic animals were incubated for 90 minutes at 33°C. These conditions cause synthesis of HSP70 in *Hydra vulgaris* [32]. Heat shock significantly increased the percentage of cells showing nuclear localization of FoxO-GFP (Figure 5B). Animals from a second transgenic line, in which ectodermal epithelial cells expressed FoxO-GFP, were subjected to the same heat shock conditions and also showed an increase in fusion protein nuclear localization (data not shown).

Finally, we asked whether the stress-associated kinase JNK promotes the nuclear localization seen in response to heat shock. Transgenic animals were treated for 24 hours with either of two inhibitors of JNK, SP600125 [58] or AS601245 [59]. Treatment with the concentration of SP600125 used here has been shown to reduce JNK activity in *Hydra* [60]. Following 24 hours of treatment, animals in inhibitor were placed at 33°C for 90 minutes to induce heat shock. When subjected to heat shock, animals treated with either of the two JNK inhibitors showed significantly less nuclear localization of FoxO-GFP than heat-shocked control animals (Figure 5C, D) These results show that in *Hydra*, as in bilaterian animals, JNK plays a role in increasing FoxO nuclear localization under conditions of thermal stress.

## Discussion

We have identified a single FoxO gene in a member of the cnidarian genus *Hydra*. FoxO genes have been found in members of early-evolving animal phyla, including sponges, placozoans, and cnidarians [25]–[27]. However, they were not detected in the genome of a member of the choanoflagellates, the sister taxon to metazoans, and have not been found in fungi or plants [25]. Thus, FoxO genes appear to have arisen in the common ancestor of animals.

*Hydra FoxO* is strongly expressed in interstitial cells in the body columns of adult animals. *Hydra* interstitial cells include multipotent stem cells which maintain populations of stinging cells, secretory cells, and neurons in the adult [39]. Existing data imply that *Hydra vulgaris* do not show senescence–increased mortality with age [61]. In other organisms, FoxO proteins can reduce damage to cells by increasing levels of antioxidant or heat shock proteins and by promoting genomic stability [6], [7]. One possibility is that FoxO functions to reduce damage to interstitial stem cells over the potentially long life of an individual *Hydra* and its asexually produced offspring. In addition, because *Hydra* germ cells are not segregated early in development and instead arise from interstitial stem cells [47], FoxO expression in interstitial cells could help to minimize damage that would affect gametes.

FoxO expression has been examined in another cnidarian, the marine hydrozoan *Clytia hemisphaerica*. However, it is not yet clear how similar cell and tissue dynamics are in these two species. *CheFoxO* in *C. hemisphaerica* is expressed throughout the polyp as well as in the larval and medusa stages of the life cycle not present in *Hydra*. As in the *Hydra* polyp, expression in both polyp and medusa is strongest in tissues characterized by cell proliferation [27].

Our results provide evidence that, as in bilaterian animals, *Hydra* FoxO transcriptional activity is negatively regulated by the PI3K/Akt/SGK pathway. Like other FoxO proteins, the predicted *H. magnipapillata* FoxO protein includes three Akt/SGK phosphorylation sites. Treatment with a PI3K inhibitor significantly increased nuclear localization of the FoxO-GFP fusion protein (Figure 5A). Our results are consistent with recently reported data which also imply that *Hydra* FoxO function is affected by the PI3K/Akt/SGK pathway. Lasi et al. [62] induced transient expression of *Hydra* FoxO fused to GFP by introducing plasmid DNA into epithelial cells using a particle gun. Approximately 20–60% of *Hydra* epithelial cells expressing FoxO-GFP underwent apoptosis [62]. An insulin/IGF-1 receptor gene [63] and three insulin-like peptide genes (unpublished information, Genebank accession numbers GU219979, GU219980, and GU219981) are present in *Hydra*. Coexpression of one of the *Hydra* insulin-like genes with the FoxO-GFP protein decreased the rate of apoptosis in epithelial cells [62]. This result provides evidence that insulin/IGF-1 signaling, which acts through the PI3K/Akt/SGK pathway, reduces *Hydra* FoxO activity. Regulation of FoxO mediated by PI3K/Akt/SGK thus appears to be conserved between *Hydra*, *Drosophila*, *C. elegans*, and mammals.

Unlike Lasi et al., we seldom observed apoptosis in FoxO-GFP expressing cells, judging by DNA distribution in DAPI-stained cells. This difference in results could be caused by differences in levels of expression of the fusion protein. It could also reflect differences between the epithelial cells examined by Lasi et al. and cells of the interstitial lineage. Because in situ hybridization showed strong *FoxO* expression in cells of the interstitial cell lineage, in most experiments we characterized FoxO-GFP localization in these cells rather than in epithelial cells.

In *Hydra*, changes in feeding rate alter growth rate. Greater food availability and higher growth rate result in larger animals and more rapid production of new buds [64]. Decreases in food availability result in slower epithelial cell division [64], [65], apoptosis of some epithelial cells [65]–[67] and autophagy in some epithelial cells [68]. FoxO proteins in other organisms can mediate responses to dietary restriction, act to slow cell cycling, and trigger apoptosis [6]. FoxO could therefore potentially be involved in the responses to dietary restriction seen in *Hydra*. If *Hydra* insulin/IGF-1 levels decrease under low nutrient conditions, we might expect to see increased FoxO-GFP nuclear localization in starved animals. Interestingly, we did not find that starvation affected FoxO-GFP localization. Our results might still be consistent with a role for FoxO in response to low nutrient conditions. In *C. elegans* and mammals, under low nutrient conditions AMP-activated protein kinase phosphorylates FoxO proteins and causes increased FoxO transcriptional activity without affecting FoxO cellular localization [5], [69].

However, our results might also reflect differences in the effects of low nutrient conditions on epithelial cells and interstitial lineage cells. Apoptosis, autophagy, and changes in cell cycle length in response to dietary restriction have been reported only for epithelial cells [64]–[68]. Experiments with chimeric animals containing epithelial cells and interstitial cells from different *Hydra* strains show that the growth rate of adult *Hydra* is determined primarily by epithelial cells [70], [71]. In contrast, rates of interstitial stem cell division and differentiation appear to be affected by interstitial cell density [53]. The *Hydra* insulin/IGF-1 receptor gene is known to be expressed in the ectoderm [63]. Under low nutrient conditions, decreased insulin/IGF-1 signaling may lead to increased FoxO function and apoptosis in epithelial cells, while our results may indicate the absence of a corresponding response to low nutrient conditions in *Hydra* interstitial cells.

In bilaterian animals, FoxO proteins play important roles in cellular resistance to stress. Our data on FoxO-GFP localization provide evidence that FoxO also mediates stress resistance in *H. vulgaris*. Conditions known to induce heat shock in *H. vulgaris* significantly increased nuclear localization of the fusion protein in both interstitial lineage cells (Figure 5B) and ectodermal cells. In other animals, JNK kinases, which are activated in response to environmental stresses including heat shock and oxidative stress, increase nuclear localization and transcriptional activity of FoxO proteins [72], [73]. A JNK gene is present in *Hydra* [74]. We found that in *Hydra vulgaris*, JNK inhibitors reduced nuclear localization of FoxO-GFP under heat shock conditions (Figures 5C,D). Our results suggest that, as in bilaterians, JNK increases FoxO activity and cellular resistance to stress in *Hydra*.

The effects of environmental stresses such as rising sea surface temperatures, pollutants, and pathogens on cnidarians, especially corals, are a matter of serious concern. Understanding the molecular mechanisms of cnidarian responses to environmental stresses is of considerable interest. Our experiments provide evidence that increased activity of FoxO proteins in response to thermal stress, as well as regulation of FoxO activity by Akt and JNK, were present in the common ancestor of cnidarians and bilaterians. Multiple recent studies have characterized stress-induced changes in the transcriptomes of cnidarian species e.g. [75]–[78]. Since much of the regulation of FoxO protein function is posttranscriptional, our results complement information from such studies. Further understanding of the role of FoxO in stress responses in *Hydra* and other cnidarians could provide insight into the details of these organisms' physiological responses to stress. Bilaterian FoxO proteins act to integrate different environmental signals, since they mediate responses to multiple stresses including low nutrient levels, oxidative stress, and thermal stress [6], [7]. Information about the roles of FoxO proteins in cnidarians may be important in understanding how cnidarian species respond to the combinations of environmental challenges many of them experience.

## Materials and Methods

### *Hydra* strains used and culture conditions

*Hydra magnipapillata* strain 105 and *Hydra vulgaris* strain AEP were maintained under standard culture conditions at 18°C. Animals were fed with *Artemia salina* (Brine Shrimp Direct) nauplii cultured from cysts for 48 hours at room temperature.

### Hydroxyurea treatment

To reduce the interstitial cell population, *H. magnipapillata* were incubated in 10 mM hydroxyurea for 24 hours and then in medium without hydroxyurea for 12 hours. This cycle was repeated two additional times [53], followed by four days of culture without hydroxyurea. Animals were fed daily during treatment. Animals were then either macerated or used for whole-mount in situ hybridization. Six treated and six control animals were macerated as described by David [54] except that to disassociate tissue into individual cells, 1.5 ml microcentrifuge tubes containing animals in maceration solution were left for 30 minutes taped to the side of a Vortex Genie mixer set at intermediate speed. Following maceration, numbers of epithelial cells, interstitial cells, nematoblasts, nematocytes, neurons, and gland cells were determined for treated and control animals.

### Isolation of *Hydra magnipapillata* FoxO and phylogenetic analysis

To identify FoxO genes, we searched the assembled *H. magnipapillata* 105 genome sequence in the Metazome database using tblastn with DAF-16 and dFOXO protein sequences as queries. DNA encoding *H. magnipapillata* FoxO was isolated from first strand cDNA by PCR using the primers GCGAGATATGTTTTTAAATGTCAGTGC and TCCATATAGAACTTTCCTGAGTTCATTAG. cDNA was produced from *H. magnipapillata* poly-A RNA using the Invitrogen GeneRacer Kit.

Phylogenetic analyses were based on the predicted amino acid sequence of the forkhead domain. Sequences were aligned using ClustalX [79], [80] with the following Multiple Alignment Parameters: Gap Opening Penalty: 10.00, Gap Extension Penalty: 0.20. The most parsimonious tree (Figure 2) was found using PAUP* 4.0 [81] implementing a full heuristic search with 10 random stepwise-addition replicates and TBR branch swapping. Amino acid substitutions were weighted using the protpars matrix of PAUP. Maximum Parsimony, Maximum Likelihood, and Neighbor Joining bootstrap values were calculated based on 1000 replicates. For Maximum Parsimony each bootstrap replicate consisted of a full heuristic search with 10 stepwise-addition random replicates and TBR branch swapping. Maximum likelihood bootstrap was implemented using the Phylip [82] programs SEQBOOT, PROML (with JTT model of amino acid change), and CONSENSE. Bayesian posterior probabilities were calculated using MrBayes version 3.1.2 [83], [84] with two independent runs of 1,000,000 generations each, sampled every 1000 generations with four chains. (Temperature was set at the default value of 0.2; 10% of the first samples were used as burnin.) The average standard deviation split frequencies after 1,000,000 generations was 0.011.

### *In situ* hybridization

A 992 base pair long portion of the *FoxO* coding sequence was amplified from *H. magnipapillata* 105 cDNA using the primers CCCAGATGCAAAAGCAGGGAAATC and GCTTTACTGGTCTAAGTCGCTCGG. This PCR product was cloned into the Promega pGEM-T Easy Vector. To produce the digoxygenin-labeled antisense probe, the plasmid was linearized by digestion with SalI and in vitro transcription was performed using the Roche DIG RNA Labeling Kit and T7 RNA polymerase. To produce the sense probe, the plasmid was linearized with NcoI and transcription was performed using the SP6 polymerase. Whole mount in situ hybridization was performed as described in [85] and [86], except that RNA probes were heated to 65°C for five minutes immediately before use.

### Transgenic *Hydra*

We produced an expression construct with the *H. magnipapillata* 105 *FoxO* coding sequence fused in frame with a sequence encoding enhanced green fluorescent protein, under the control of the *Hydra* β-actin promoter. Using PCR, an NheI site was added to the 5′ end of the *FoxO* cDNA and a HpaI site was added to the 3′ end. The resulting cDNA was cut with NheI and HpaI, and then cloned into the expression vector pHyVec4 (GenBank accession DQ385853), which had been cut with NheI and SmaI. pHyVec4 is a modified version of the hoT G plasmid [31]. The structure of the construct was confirmed by DNA sequencing. The *FoxO* coding sequence within the construct was checked against the NCBI trace archive sequences for *H. magnipapillata* 105. Plasmid DNA for embryo injection was purified using the Qiagen Endo-free Gigaprep kit and resuspended in sterile deionized water.

The FoxO-GFP expression construct was microinjected into embryos of *H. vulgaris* strain AEP at the one to eight cell stage as described in Wittlieb et al. [31], using a Narishige IM 300 microinjector. Needles for injection were produced using a Sutter P-30 micropipette puller. Each of the three stably transgenic animals produced was propagated through budding to produce a line of transgenic *Hydra*. The majority of experiments were conducted with a line termed in[act:FoxO-GFP]1, in which a subset of cells of the interstitial lineage expressed the fusion protein. Specifically, the cells which expressed the protein were precursors to stenotele nematocytes, mature stenotele nematocytes, and ganglionic neurons. Fusion protein localization was examined in all of these cell types. The number of cells of each cell type scored was approximately equal in the control and treated animals in each experiment. Fusion protein localization following heat shock was also examined in the line ec[act:FoxO-GFP]1, in which ectodermal cells expressed the fusion protein.

### Heat shock, inhibitor and starvation treatments

Animals subjected to heat shock were incubated at 33°C for 90 minutes, conditions known to increase levels of HSP70 [32]. Animals treated with the PI3K inhibitor LY294002 (Sigma) were incubated for 1 hour in the dark at 18°C in 40 µM LY294002 in hydra medium containing 0.2% ethanol. Animals treated with the JNK inhibitor SP60025 (Calbiochem) were incubated in the dark in 2.5 µM SP60025 in hydra medium containing 0.0125% DMSO. Animals were first incubated for 24 hours at 18°C and then placed at 33°C for 90 minutes. Control animals were incubated at the same temperatures in N^1^-Methyl-1,9-pyrazoloanthrone (Calbiochem) in hydra medium containing 0.0125% DSMO. Animals treated with the JNK inhibitor AS601245 (Calbiochem) were incubated in the dark in 2 uM AS601245 in hydra medium containing 0.15% DMSO. They were first incubated for 24 hours at 18°C and then placed at 33°C for 90 minutes. Control animals were incubated at the same temperatures in 0.15% DSMO/hydra medium. Animals subjected to starvation were fed daily for two weeks and then starved for two, three, four, or ten days. Control animals were fed daily but starved for one day before fixation to reduce background fluorescence. In heat shock, LY294002, SP60025, four-day starvation and ten-day starvation experiments, three treated and three control *Hydra* were used for each experiment. In AS601245, two-day starvation and three-day starvation experiments, five treated and five control *Hydra* were used. For all treatments, the percentage of cells showing nuclear localization in control and treated animals were compared using the Mann-Whitney U test.

### Microscopy

To determine FoxO-GFP localization, animals were processed as follows. Following experimental treatments, animals were fixed for 1 hour in 4% paraformaldehyde in hydra medium and then washed for 10 minutes in PBS, for 30 minutes in 5 µg/mL DAPI in PBS, and for 5 minutes in PBS. Fixation and washes were performed at 4°C. Cells expressing the fusion protein were visualized at 1000× using a Nikon Eclipse 80i microscope and photographed using a SPOT RTke digital camera. Localization was examined in nematoblasts, nematocytes, and neurons.
